# Model of Processive Catalysis with Site Clustering and Blocking and Its Application to Cellulose Hydrolysis

**DOI:** 10.1021/acs.jpcb.2c05956

**Published:** 2022-10-17

**Authors:** Zdeněk Petrášek, Bernd Nidetzky

**Affiliations:** † Institute of Biotechnology and Biochemical Engineering, Graz University of Technology, NAWI Graz, Petersgasse 12, A-8010Graz, Austria; ‡ Austrian Centre of Industrial Biotechnology, Petersgasse 14, A-8010Graz, Austria

## Abstract

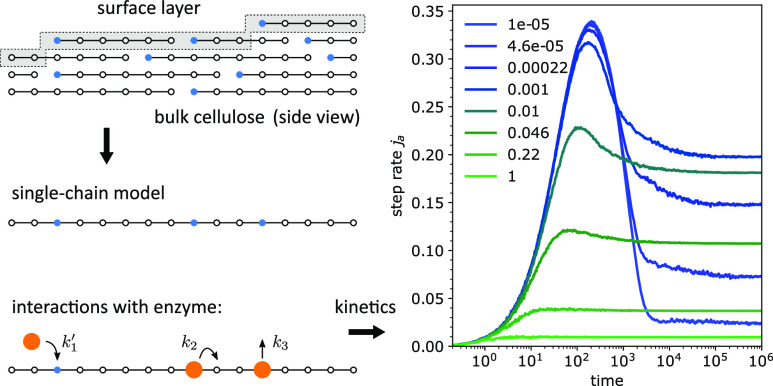

Interactions between particles moving on a linear track and their possible blocking by obstacles can lead to crowding, impeding the particles’ transport kinetics. When the particles are enzymes processively catalyzing a reaction along a linear polymeric substrate, these crowding and blocking effects may substantially reduce the overall catalytic rate. Cellulose hydrolysis by exocellulases processively moving along cellulose chains assembled into insoluble cellulose particles is an example of such a catalytic transport process. The details of the kinetics of cellulose hydrolysis and the causes of the often observed reduction of hydrolysis rate over time are not yet fully understood. Crowding and blocking of enzyme particles are thought to be one of the important factors affecting the cellulose hydrolysis, but its exact role and mechanism are not clear. Here, we introduce a simple model based on an elementary transport process that incorporates the crowding and blocking effects in a straightforward way. This is achieved by making a distinction between binding and non-binding sites on the chain. The model reproduces a range of experimental results, mainly related to the early phase of cellulose hydrolysis. Our results indicate that the combined effects of clustering of binding sites together with the occupancy pattern of these sites by the enzyme molecules play a decisive role in the overall kinetics of cellulose hydrolysis. It is suggested that periodic desorption and rebinding of enzyme molecules could be a basis of a strategy to partially counter the clustering of and blocking by the binding sites and so enhance the rate of cellulose hydrolysis. The general nature of the model means that it could be applicable also to other transport processes that make a distinction between binding and non-binding sites, where crowding and blocking are expected to be relevant.

## Introduction

Transport of independent particles moving at high densities along a linear track is often slowed down by their mutual interactions.^1^ These crowding effects play an important role in many transport processes across widely different disciplines, on spatial scales ranging from the molecular nanoscale to car traffic. Examples include processive catalysis^2^ in chemistry and biochemistry, such as enzymes processively catalyzing conversion of an insoluble polymeric substrate (cellulose, chitin, or other polysaccharides),^3−6^ directional sliding of enzymes along a nucleic acid in processes such as DNA polymerization^7^ and protein synthesis,^8^ active cellular transport phenomena, such as motor proteins walking along microtubules^9^ and actin filaments,^10^ but also problems in seemingly unrelated fields, for example, movement of ants along a trail in ecology studies^11^ and the formation of traffic jams in car traffic.^12,13^

The spatial correlations inevitably present in these processes mean that the particles cannot be described as a priori fully independent actors, as, for example, reactants in bulk chemical kinetics. This fact complicates the theoretical description of the crowding phenomena. On the other hand, the recognition that the particular details of the underlying transport process are often not decisive for the general effects of crowding led to the development and the detailed study of the properties of rather general, abstract models.

One of the simplest models of transport affected by crowding is the totally asymmetric simple exclusion process (TASEP).^14−16^ Particles step with a constant rate in one direction along a linear chain of sites; a step is possible only if the next site is not occupied. The particle current depends on the occupation density: the maximum is reached when half of the sites are occupied; at higher occupancies, a further increase of the current due to the particle number is outweighed by the strong effect of crowding. Despite its simplicity, the model can predict complex stationary states, for example, a state with two regions of different particle densities separated by a steep wall as a result of boundary conditions in a system with open boundaries.^7^ In one of its first applications, this model has been used to describe the kinetics of ribosomes moving along mRNA.^7^

In a further development, the model was extended by including the exchange of particles with the bulk reservoir by considering binding to and detachment from the chain (Langmuir kinetics, LK), leading to the TASEP-LK model.^17^ Later, variations of the model with modified exchange rules or interactions between particles moving along different chains have been introduced and studied.^18,19^ Although many works deal with the steady states of systems with open boundaries, where the boundary condition strongly influences the steady state, in some cases, relaxation kinetics^19,20^ or systems with periodic boundary conditions^18−20^ have been investigated. The exchange of particles with the bulk is particularly relevant for chemical and biological applications. Models based on TASEP-LK have often been applied to the description of crowding dynamics of the motor proteins moving along the filaments of cytoskeletal networks.^9,21,22^

The models of transport processes are closely related to the models of crystal growth and evaporation.^23−25^ For example, the role of impurities in crystal growth may be analogous to the effects of external obstacles in linear transport. The models of crystal growth can thus provide inspiration for formulating models of processive catalysis.

Cellulose hydrolysis by processive cellulases can be viewed as a directional transport process where crowding cannot be neglected.^3^ Crowding of cellulases while moving along the cellulose chains has been identified as one of the possible factors severely limiting the efficiency of cellulose hydrolysis.^26,27^ Cellulose on the nanoscale consists of tightly packed, oriented linear chains (polymeric cellobiose) resulting in a regular, crystalline structure. On a larger scale, these microfibrils are assembled into more or less filamentous nano- or microparticles or larger, structured composite bodies. Cellulose is insoluble in water but can be degraded down to its constituent monomers by enzymes—cellulases. Some cellulases processively and directionally depolymerize the cellulose chain while releasing cellobiose units into the bulk solution.^3^

To understand the limitations of the processive cellulose hydrolysis, a range of models have been developed. A large group of these are the kinetic models based on ordinary differential equations, formulated in analogy to the chemical enzyme kinetics.^28−30^ Although some of them include processivity of the enzyme,^31^ no spatial correlations, apart from simple blocking by a fixed obstacle, are included. Therefore, the crowding effects are not properly represented in these models, limiting their applicability.

The spatial correlations, therefore also the crowding effects, are fully included in a class of models based on simulations.^32−35^ The simulation models are typically formulated to a high level of detail. Often, they consider the size, the shape, and even the internal structure (two domains) of the enzyme^33−35^ and a specific shape of the substrate.^32^ This raises a question of how general the results of such simulations are, and to which extent they depend on the particular choice of these detailed parameters. Furthermore, a large number of variable parameters, together with the high time demand to perform the simulations, precludes an extensive exploration of the relevant parameter space and therefore a broad characterization of the model.

Here, we extend the TASEP-LK model by differentiating between binding and non-binding sites on the chain. This modification is motivated by the structure of cellulose. As a result, we obtain a minimal model showing how blocking due to crowding might affect cellulose hydrolysis. While omitting the less relevant details, only the essential features of the hydrolysis process are retained: binding to a particular end site of the oriented cellulose chain, processive motion along the chain accompanied by hydrolysis, blockage by other chain ends or enzyme particles, and detachment from the chain.

We investigate the general behavior of the model and its applicability and limitations for the description of cellulose hydrolysis. By including only one additional parameter compared to the TASEP-LK model, the modified model remains very general and thus applicable to the description of crowding effects also in other systems that make a distinction between binding and non-binding sites.

## Model

The model is inspired by an idealized picture of the hydrolysis of crystalline cellulose by the processive enzyme Cel7A.^3,36^ The parallel cellulose chains form a 3D crystal, with only the exposed surface being accessible to the enzyme particles.

**Figure 1:**
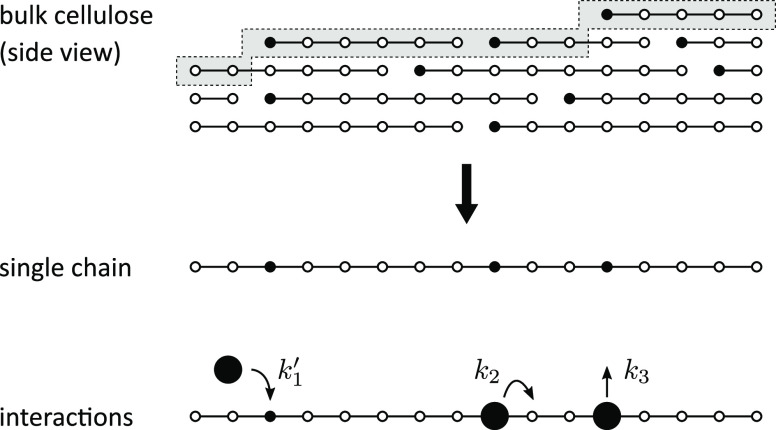
Particles can interact only with the surface of the bulk material. One lane of the surface layer is approximated with a linear chain of sites. Some of the sites allow particle attachment (binding sites, filled circles), others do not (non-binding sites, empty circles). The particles interact with the chain in three ways: attachment to the binding sites with the rate *k*_1_^′^; making a step to the site on the right (allowed only if the site is non-binding) with the rate *k*_2_; and detachment with the rate *k*_3_.

The cellulose chains are oriented and the particles (enzyme molecules) can bind to only one end site of the chain (the left chain end site in Figure 1). Once attached, the enzyme steps along the chain from site to site (from the left to the right in Figure 1), removing one segment per step (hydrolyzing the glycosidic bond of the cellulose chain and releasing a cellobiose molecule) until it either detaches, or becomes blocked by an end of a chain lying in the layer above it, or reaches the end of the chain, in which case we may assume that it detaches.

To further simplify this situation, we model one lane in the upper exposed surface of the cellulose crystal as a single chain with distributed binding sites (Figure 1). This reduction to a one-dimensional chain neglects the loss of binding sites by reaching the chain end (complete chain hydrolysis) and ignores the exposure of new binding sites as the chain is consumed. However, these two features compensate each other in a sense that the total number of binding sites is preserved on average. Importantly, the essential feature of this model—the blockage of the stepping particle by binding sites in front of it—is preserved.

The linear chain consists of *N* sites of two types: binding sites, to which particles from the reservoir can bind, and non-binding sites, which cannot directly bind a particle from the reservoir but can become occupied by a particle stepping in from the neighboring site. The fraction of the binding sites is denoted as *u*.

The particles can bind to the binding sites with a rate *k*_1_^′^, step to the neighboring site with a rate *k*_2_ (while releasing a product molecule), and detach with a rate *k*_3_ (Figure 1). The stepping process is totally asymmetric; the particles move from the left to the right. While making steps, the particle “carries” the binding site with it: the site from which the particle steps out becomes non-binding, the site to which a particle steps becomes binding, and the site from which the particle detaches remains a binding site. The particle can make a step only if the next site is non-binding, that is, binding sites, regardless of whether occupied or not, cannot be entered by making a step from the neighboring site. This blocking by binding sites is the origin of the observed crowding effects.

The model has four independent parameters: the fraction of the binding sites *u* and the rates of attachment *k*_1_^′^, stepping *k*_2_, and detachment *k*_3_. We further distinguish between two cases depending on the number of particles in the reservoir available for binding. In the first case, the total number of particles *e*_0_ is assumed to be in excess of the total number of binding sites *uN*: *e*_0_ ≫ *uN*, so that the number of free particles available for binding *n*_e_ can be approximated by the total number of particles *e*_0_: *n*_e_ = *e*_0_ – *n*_a_ > *e*_0_ – *uN* ≈ *e*_0_, where *n*_a_ is the number of bound particles. The attachment is then described by a simple rate constant *k*_1_^′^. In the second case, the total number of available particles *e*_0_ is finite, and the attachment rate at any moment is *k*_1_*n*_e_, where *k*_1_ is the corresponding rate constant. The first case can be seen as a limit of the second case when *e*_0_ ≫ *uN* and *k*_1_*e*_0_ = *k*_1_^′^.

In all simulations, a periodic boundary condition was applied. The initial distribution of the binding sites was in most cases chosen as random, but the simulations with removal and reattachment of all particles can be interpreted as a sequence of independent simulations with modified initial conditions corresponding to a variable degree of initial clustering of binding sites.

In the current model with one chain and a periodic boundary condition, the amount of substrate available for conversion is effectively infinite. This is intentional, as we are focused on the effects of crowding on the step rate isolated from other effects, such as substrate depletion. The effect of substrate depletion could, however, be easily included in the model by monitoring in the course of simulation how many times a particular site on the chain is visited by a stepping particle. Each passing of a site on the chain would correspond to the conversion of one layer of the substrate. Setting the maximum number of layers would allow modeling of substrates with different particle sizes and observation of the substrate depletion effects.

In the following, a “cluster” refers to one or more binding sites next to each other, regardless of their occupancy, a “gap” is a sequence of non-binding sites between two clusters, and an attached particle is called blocked when it cannot make a step because its neighboring site (to the right) is a binding site, regardless of whether occupied or not.

Because clustering of the binding sites and blocking of the stepping particles turns out to be decisive for the particles kinetics, we refer to the proposed model as the CB model (clustering/crowding + blocking).

## Methods

The model was implemented as a simulation in Python (ver. 3.6), taking advantage of parallel computation on a CUDA graphics card provided in Python via the Numba compiler. The chain parameters were chosen to match the internal architecture of the GPU and so to optimize the calculation speed.

The chain length was *N* = 8192 sites, which is long enough to avoid any artificial effects due to the periodic boundary condition. The simulation was performed in parallel on 128 segments of the chain, each 64 sites long, and on 24 chains simultaneously. To increase the signal-to-noise ratio, the simulations were repeated 10–100 (in some cases up to 2000) times, representing an average of over 240–2400 chains.

In every simulation step, the particles attached to the chain and not blocked were tested for the possibility of making a step with the rate *k*_2_ and for detachment with the rate *k*_3_. The unoccupied binding sites were tested for the possibility of attachment of a particle from the bulk with the rate *k*_1_^′^. After all steps, attachments and detachments within the current simulation step were performed, the number of particle steps, particle attachments and detachments per chain, the number and size of clusters, and other parameters were stored for further analysis.

The value of the step rate *k*_2_ was fixed to *k*_2_ = 1 in all simulations. The attachment and detachment rates used in the simulations were varied in the following ranges: *k*_1_^′^ = 10^–4^ to 0.1, *k*_3_ = 10^–5^ to 1.0. The dimensionless rates used in simulations can be related to the corresponding experimental rate constants (indicated with a star here) as follows:  and *k*_3_/*k*_2_ = *k*_3_^*^/*k*_2_^*^. In the case when the particles are not in excess of the number of binding sites, we have two additional expressions: *k*_1_*e*_0_/*k*_2_ = *k*_1_^*^c_tot_/*k*_2_^*^ and *e*_0_/(*uN*) = *c*_tot_/*c*_s_, where the second one relates the total numbers of particles in simulations *e*_0_ and of binding sites on the chain *uN* to the total volume concentrations of the enzyme *c*_tot_ and accessible binding sites on the substrate *c*_s_. Using these expressions, it is possible to directly convert the experimental parameters to the dimensionless parameters used in simulations while choosing *k*_2_ = 1. To perform the conversion in the opposite direction, from the simulation parameters to the real-world dimensional parameters, it is necessary to provide two parameters that provide scaling for time and concentration, for example, the step rate constant *k*_2_^*^ and the total enzyme concentration *c*_tot_.

The experimentally determined rates are generally spread over broad ranges, depending on exact conditions and substrate type. The following values are typically reported:^37^ the step (hydrolysis) rate constant *k*_2_^*^ = 2–10 s^–1^, the attachment rate constant *k*_1_^*^ = 0.003–0.3 μM^–1^ s^–1^, with the employed concentration of cellulase *c*_tot_ = 0.1–2 μM,^31,38^ and the detachment rate constant *k*_3_^*^: 0.002–0.2 s^–1^. The value ranges of the dimensionless rates *k*_1_^′^ and *k*_3_ used in the simulations therefore cover the ranges of the experimentally determined values.

## Results

First, we show the analysis of the binding kinetics, as it allows analytical description due to its independence on the neighborhood of the binding site. Then, the results of simulations of particles binding to, detaching from, and stepping along the chain are presented. This part is divided into two sections depending on whether the number of available particles is in excess of the binding sites or not.

### Binding Kinetics

In the presented model, the binding kinetics are independent of the stepping of the attached particle and the occupancy of the neighboring sites. Therefore, the binding kinetics can be described without considering the spatial distribution of the binding sites, and no effects of spatial correlations are present.

The attachment of free particles *E* to the unoccupied binding sites *F* and the detachment of the bound particles *A* can be described by the following reaction scheme:
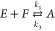
1with the attachment and detachment rate constants *k*_1_ and *k*_3_, respectively. The numbers of free particles *n*_e_, unoccupied sites *n*_f_, and occupied sites (attached particles) *n*_a_ are related by the conservation relations *n*_e_ + *n*_a_ = *e*_0_ and *n*_f_ + *n*_a_ = *uN*.

The temporal evolution of the number of bound particles is governed by the following differential equation
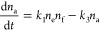
2

Instead of *n*_a_(*t*), we choose to describe the system by the fraction of occupied binding sites *f*(*t*), and by substitutions *n*_f_ = (1 – *f*)*uN* and *n*_a_ = *fuN* we obtain
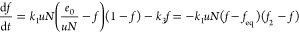
3

The equilibrium fraction of occupied binding sites *f*_eq_ is obtained as the smaller of the two solutions of the quadratic equation obtained by setting d*f*/d*t* = 0 in eq 3
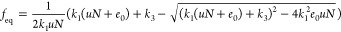
4We denote *f*_2_ the second root of the quadratic equation (*f*_2_ > 1 ≥ *f*_eq_ > 0).

The solution of eq 3 for the initial condition with no attached particles *f*(0) = 0 is
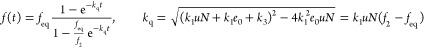
5

In the limiting case of particle excess (*e*_0_ ≫ *uN*, *k*_1_*e*_0_ = *k*_1_^′^), the solution converges to a simple exponential kinetics with the rate equal to the sum of the attachment and detachment rate constants *k*_1_^′^ and *k*_3_
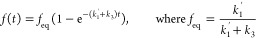
6

### Excess of Particles

The interplay between particle binding and detachment and its stepping kinetics leads to spatial correlations in the distribution of binding sites, which present a major obstacle to describing the system by a kinetic model based on differential equations. For this reason, we implemented the model as a particle-based simulation.

The initial distribution of binding sites on the chain was chosen as random. The simulations were started with particles initially attached to randomly selected binding sites. The number of particles was the same as the binding equilibrium value (eq 6). In this way, the mean number of attached particles was constant all the time, and any effects of the variation of the mean attached particle numbers were eliminated; only the blocking and clustering effect was responsible for the observed changes in the step rate.

**Figure 2:**
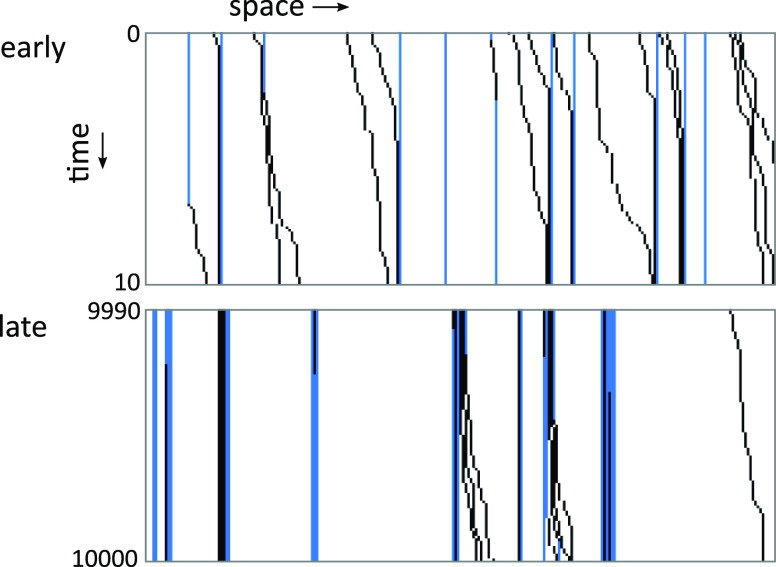
State of the chain at early and late times of the simulation. At early times, the binding sites are randomly distributed and not clustered, and many stepping (not blocked) particles are present. At late times, the binding sites are clustered, many particles are blocked, and only few particles are free to step. The stepping direction is from the left to the right (blue: unoccupied binding sites; black: occupied binding sites; white: non-attachable sites). Only 250 sites of the whole chain (8192 sites) are shown.

Figure 2 shows a graphical representation of the chain with attached and stepping particles at the beginning of the simulation and at late times. At early times, the binding sites are still randomly distributed and not clustered, and many non-blocked particles stepping from the left to the right can be observed. At late times, the binding sites are clustered, many particles are blocked, and only few particles are free to step. Notice that an attachment of a particle to the rightmost site of a partially occupied cluster may result in a micro-burst of activity, whereby several blocked particles are freed practically simultaneously. This can be related to a partial dissolution of clusters of enzyme molecules bound to a cellulose fiber, which has been previously observed experimentally (Movie S5 in ref (27)).

The temporal evolution of several parameters was extracted from the simulations: the step rate (the current) per site *j*, the mean cluster size *s*, the number of attached particles *n*_a_, and the fraction of blocked particles *f*_b_.

In the simulations with initially attached particles at their equilibrium number *n*_a_ = *f*_eq_*uN*, the mean number of attached particles naturally remained constant throughout the simulation. When the occupancy and the blocked state of the site are uncorrelated, the fraction of blocked particles *f*_b_ is directly related to the step rate *j*
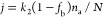
7as only the non-blocked particles contribute to the step rate at any time. Therefore, with this initial condition, two of the monitored parameters are of the highest interest: the step rate per site *j* and the mean cluster size *s*.

The step rate per site *j* is in this section displayed normalized as the step rate per attached particle *j*_p_ = *jN*/*n*_a_. It therefore directly represents the average reduction in step rate that an attached particle experiences due to the crowding effects. Because the mean number of attached particles *n*_a_ does not change throughout the simulation, *j*_p_ is directly proportional to the step rate per site *j* and also to the step rate per attachable site *j*_a_ = *j*/*u* used in the following section, when the simulation is started without any attached particles.

#### Step Rate Decrease

**Figure 3:**
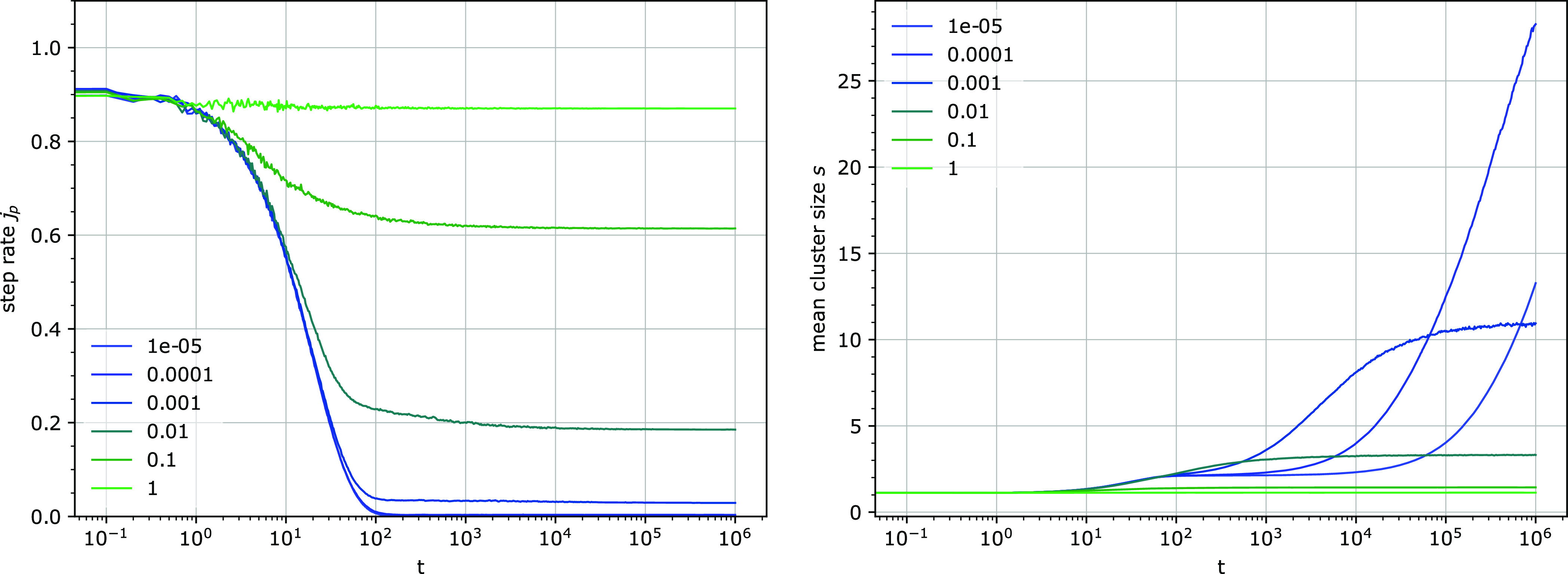
Temporal evolution of the step rate per attached particle *j*_p_ (left) and the mean cluster size *s* (right) for several attachment (*k*_1_^′^) and detachment rates (*k*_3_) with a constant fraction of occupied binding sites *f*. Other parameters: *k*_1_^′^ = *k*_3_, *u* = 0.1.

**Figure 4:**
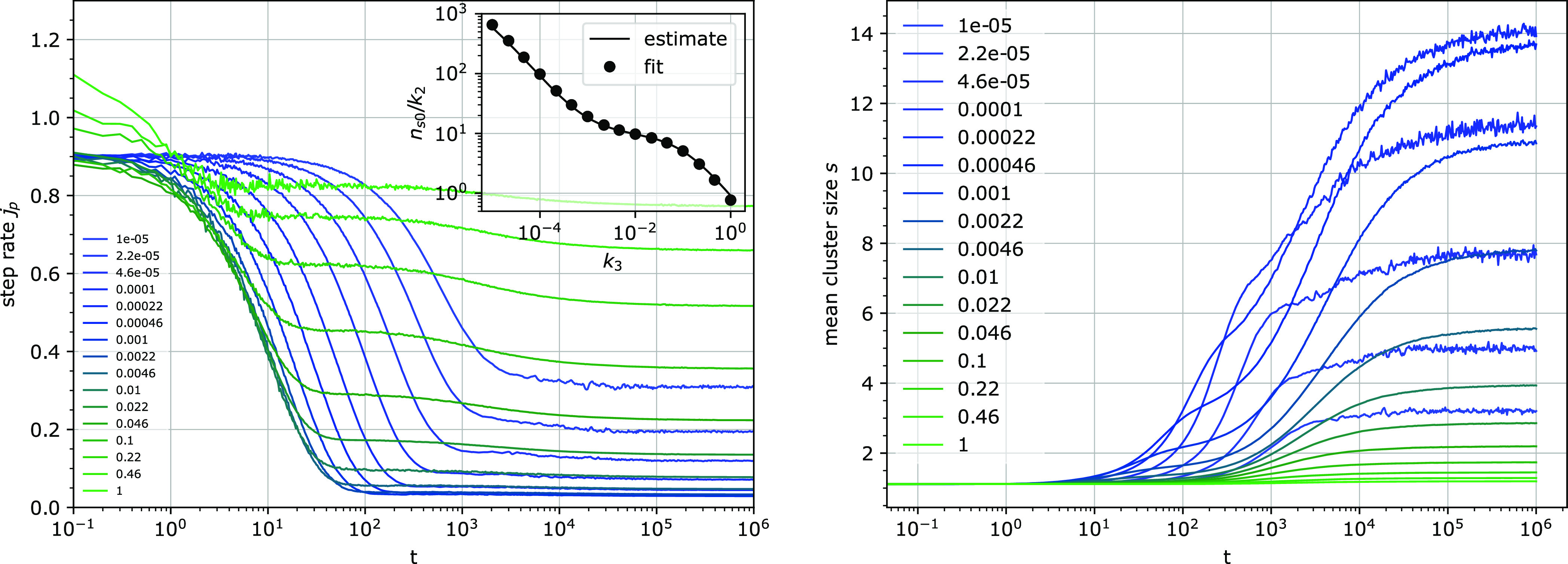
Step rate per attached particle *j*_p_ (left) and the mean cluster size *s* (right) for several detachment rates *k*_3_. The inset on the left shows the estimate of the time scale of the initial decrease of the step rate *j*_p_ (eq 8) compared with values obtained from exponential fits of the simulation data. Other parameters: *k*_1_^′^ = 0.001, *u* = 0.1.

The most dominant effect, observed to some degree in practically all simulations, is the decrease of the step rate per particle *j*_p_ from its initial value *j*_p_(0) = *k*_2_(1 – *u*) to a steady-state value, which depends on the rates *k*_1_^′^ and *k*_3_ and on the fraction of binding sites *u* (Figures 3 and 4).

Two phases of this decrease can be discerned. The first, major phase originates from the initially non-blocked particles stepping before they either become blocked by reaching the next binding site or before they detach from the chain. The time scale of this phase can be estimated as *n*_s0_/*k*_2_, where *n*_s0_ is the mean length of a free run (in steps). The inverse of *n*_s0_, the “stopping rate”” per particle step, can be approximated by the sum of the blocking rate per step 1/*s*_g_ and the detachment rate per step *k*_3_/*k*_2_. The blocking rate is the rate at which the particle becomes blocked, and *s*_g_ is the mean size of the gap between two clusters (*s*_g_ = *s*(1/*u* – 1) in general, which becomes *s*_g_ = 1/*u* for an initially random distribution of binding sites). This approximation is valid when the mean occupancy of binding sites *f* is small. When the probability of a binding site being occupied is high, the mean number of steps before a stepping particle becomes blocked is higher than the mean gap size *s*_g_ because the site on the right site of the gap can move further to the right, if occupied. Considering different cluster sizes on the right side of the gap and their probability of being fully occupied leads to the extension of the covered distance before becoming blocked from *s*_g_ to *s*_g_ + *n*_s0_*f*(1 – *u*)/(1 – *uf*). Then
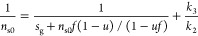
8and *n*_s0_ can be obtained as a solution of a quadratic equation. Within the probed range of simulation parameters, there is a good agreement between this estimate and the time constant obtained from an exponential fit to the initial decay of *j*_p_(*t*) in the simulation results (inset in Figure 4).

The second, weaker phase of the decrease of *j*_p_ is only sometimes discernible. The time of its appearance is correlated to the second, often major increase in the mean cluster size described below.

**Figure 5:**
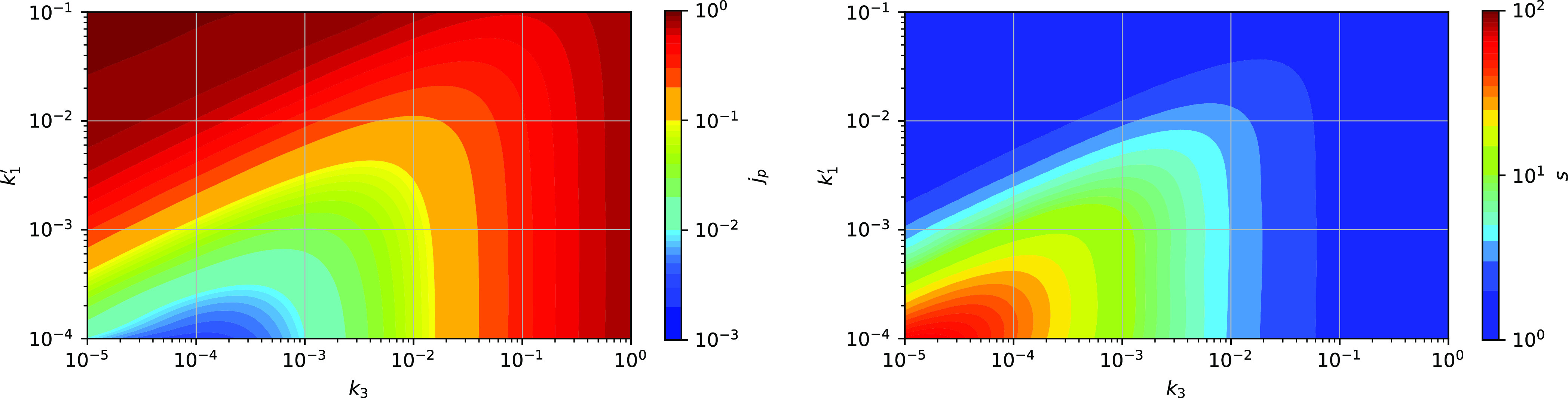
Steady-state step rate *j*_p_ (left) and the mean cluster size *s* (right) for a range of attachment and detachment rates (*k*_1_^′^, *k*_3_). Other parameters: *u* = 0.1.

The steady-state value of *j*_p_ depends in a complex way on the attachment and detachment rates *k*_1_ and *k*_3_ (Figure 5). In general, a high detachment rate leads to high *j*_p_ because the particle spends a short time in the blocked state. High attachment and low detachment rates also lead to a high *j*_p_ because of high occupancy of binding sites and therefore low number of immobile blocking sites. For a given attachment rate *k*_1_, there is a detachment rate *k*_3_ where the overall blocking effect of the binding sites is strongest, and therefore a minimum of *j*_p_ is reached.

#### Clustering of Binding Sites

Concurrently with the decrease of the step rate, the mean size of the clusters *s* of the binding sites increases from its initial value, which is *s*(0) = 1/(1 – *u*) for randomly distributed binding sites, and eventually reaches a steady state (Figures 3 and 4).

For a broad range of parameters, two phases in the increase of the mean cluster size can be discerned, approximately coinciding with the two phases of step rate decrease. The second phase appears to be slower than exponential, and often dominates. Interestingly, the often strong increase of clustering in this second phase is usually accompanied by only a minor decrease of the step rate.

The steady-state values of *s* depend on *k*_1_ and *k*_3_ in a way similar to *j*_p_ (Figure 5). At high *k*_3_, clustering is weaker because the stepping particle is more likely to detach before it becomes blocked and thereby contributes to the cluster growth. At high *k*_1_ and low *k*_3_, the site occupancy is high and therefore the number of immobile blocking sites is low. This leads to high dynamics preventing formation of large clusters. For a fixed *k*_1_, a value of *k*_3_ exists where these two declustering mechanisms are weakest and where *s* reaches its maximum. Increasing *k*_3_ weakens the formation of larger clusters by attachment of particles from the left; decreasing *k*_3_ enhances the cluster disassembly from the right due to the high occupancy of binding sites, including the rightmost cluster site.

Although the step rate decreases and the mean cluster size increases in the course of simulation (Figures 3 and 4), and the dependence of their steady state on *k*_1_ and *k*_3_ is similar (Figure 5), the two values are not fully correlated, and no direct universal relationship between the step rate per particle *j*_p_ and the mean cluster size *s* could be inferred. For example, for a fixed *k*_1_^′^, the minimum of the step rate *j*_p_ and the maximum of the mean cluster size *s* are reached at different detachment rates *k*_3_ (Figure 5). Considering only the steady-state values, the step rate and the mean cluster size in the performed simulations were constrained by the following relation: *s*^–2^ < *j*_p_/*k*_2_ < *s*^–1^. However, before a steady state was reached, transient values *j*_p_/*k*_2_ < *s*^–2^ were observed, indicating a complex relationship between *j*_p_ and *s*. The mean cluster size *s* alone therefore cannot be used as an indicator of the step rate *j*_p_.

The decrease of the step rate *j*_p_ and the increase of the mean cluster size *s* are prominent when the intrinsic processivity (the average number of steps the particle would perform before detachment if not hindered by any obstacle, *k*_2_/*k*_3_) is larger than the mean size of the gap between the clusters *s*_g_. In the opposite case, there is only a small decrease of the step rate and a small increase in the mean cluster size (Figure 3, the simulations with the highest *k*_3_ values). Even though the mean gap size increases with time, it has not been observed to approach or exceed the intrinsic processivity, unless already comparable to it or larger at the start of the simulation.

#### Removal and Reattachment of Particles

The simulations described so far were started in a state where the binding sites were randomly distributed along the chain, and the particles were attached at randomly selected binding sites at a concentration corresponding to the binding equilibrium. In the following, the simulations were stopped at constant time intervals, the attached particles were removed and reattached again at random binding sites, and the simulation was restarted. Because the clustering increases in the course of simulation, every re-start of the simulation effectively corresponds to a new simulation with a different initial condition. Later re-starts therefore represent an initial condition with a higher degree of binding site clustering.

**Figure 6:**
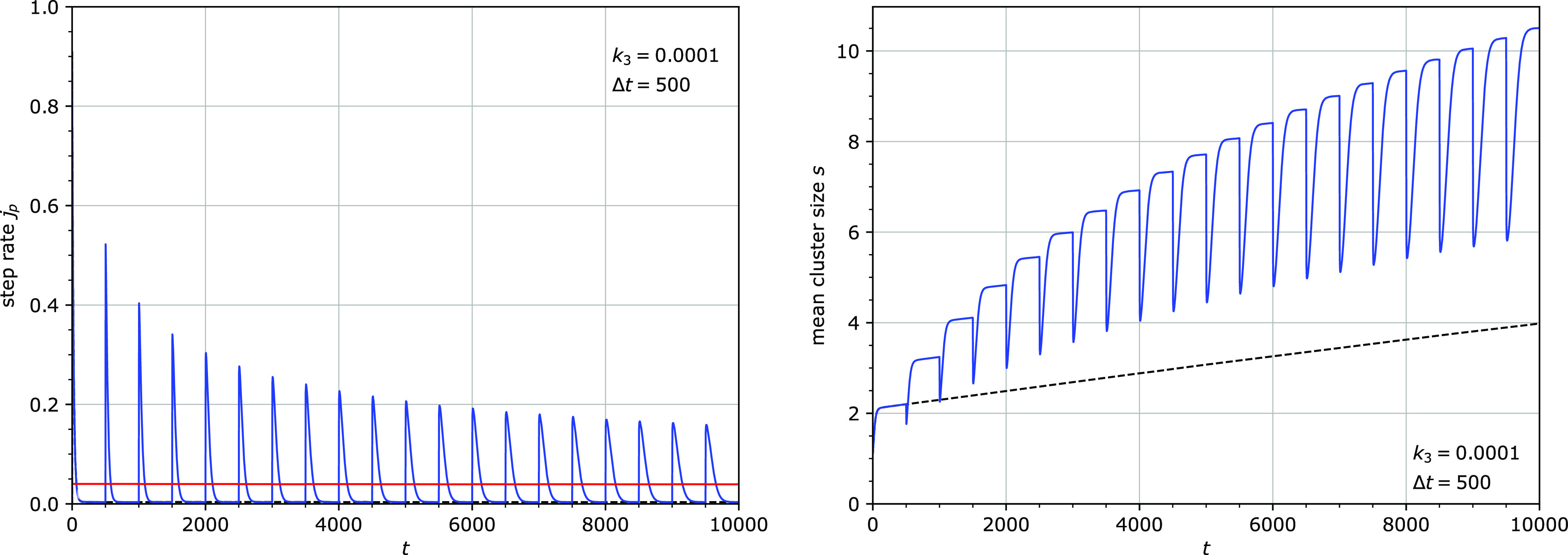
Step rate *j*_p_ (left) and mean cluster size *s* (right) for *k*_1_^′^ = *k*_3_ = 0.0001. All particles were removed and reattached at regular intervals Δ*t* = 500. The red line (left) is the mean step rate over every cycle. The dashed line (right) is the mean cluster size without periodic removal and reattachment of particles. Other parameters: *u* = 0.1.

**Figure 7:**
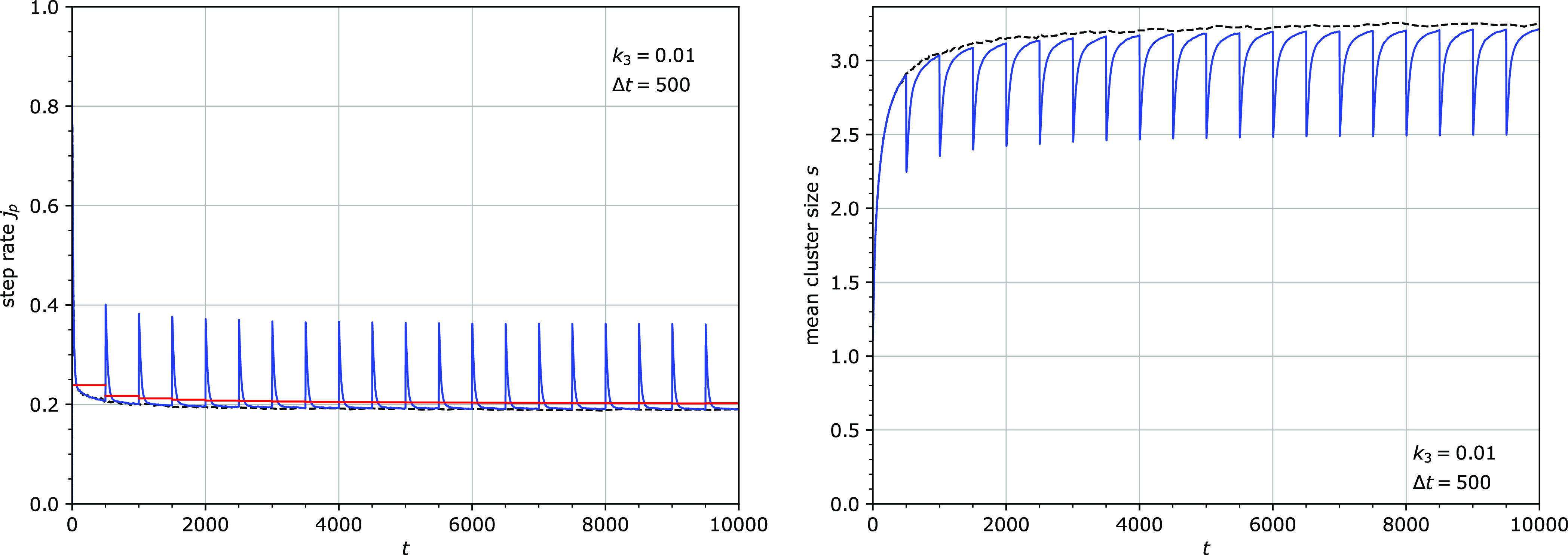
Step rate *j*_p_ (left) and the mean cluster size *s* (right) for *k*_1_^′^ = *k*_3_ = 0.01. All particles were removed and reattached at regular intervals Δ*t* = 500. The red line (left) is the mean step rate over every cycle. The dashed line (right) is the mean cluster size without periodic removal and reattachment of particles. Other parameters: *u* = 0.1.

**Figure 8:**
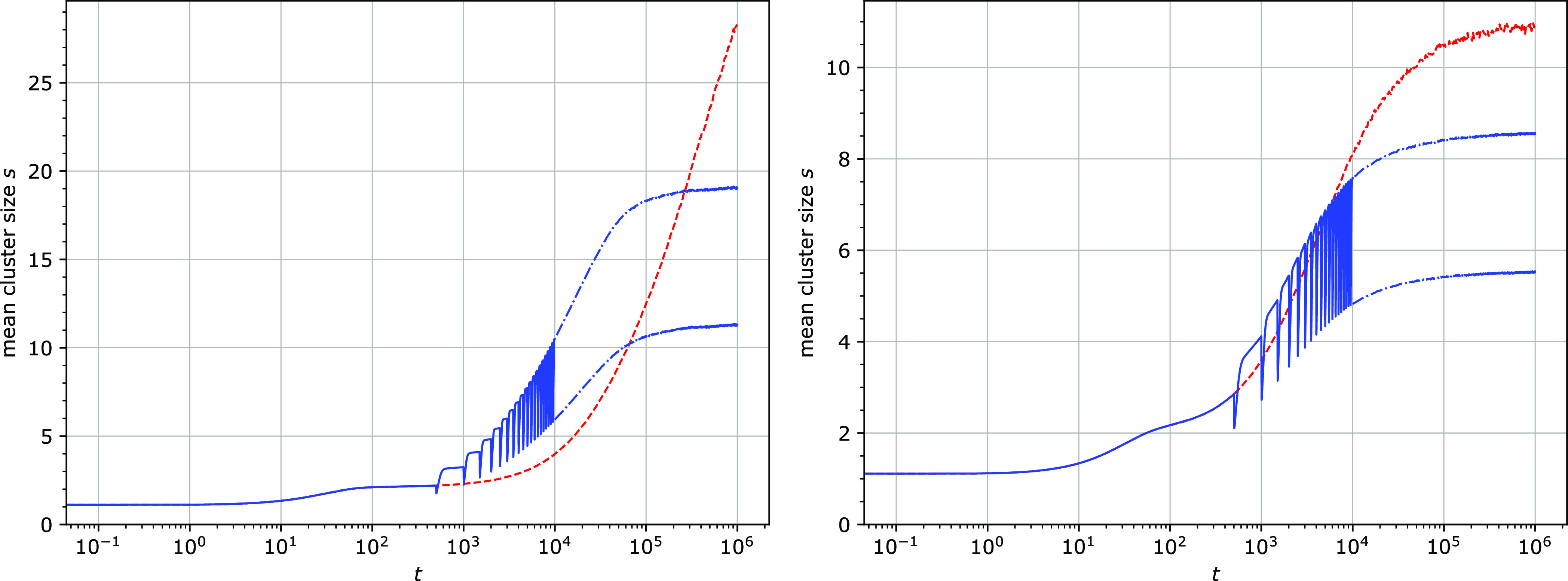
Mean cluster size *s* for two different attachment (*k*_1_^′^) and detachment rates (*k*_3_): *k*_1_^′^ = *k*_3_ = 10^–4^ (left) and 10^–3^ (right). All particles were removed and reattached at regular intervals Δ*t* = 500. The red dashed lines show the mean cluster size in the absence of the detachment/reattachment cycles. For the times *t* > 10^4^, only the minimum and maximum values per cycle are shown. Other parameters: *u* = 0.1.

Removal and reattachment of the particles were found to result in a temporary increase of the step rate. The shape of this transient burst in step rate varied depending on the state of the chain: at longer times, when the sites were more clustered, the bursts were lower and broader. The bursts were present even at very long times, when a quasi-steady state was reached, a state when the burst shape does not change any more (Figures 6–8).

The periodic removal and reattachment also led to an increase in the average step rate. The strength of this effect, however, depends on the frequency of removal and reattachment. The effect is significant only when the period of the detachment/attachment cycle Δ*t* was smaller than the inverse of the particle detachment rate *k*_3_: Δ*t* < 1/*k*_3_. Examples of the strong and the weak effects are shown in Figure 6, where the cycle period of 500 is smaller than 1/*k*_3_ = 10^4^, and in Figure 7, where the cycle period of 500 is larger than 1/*k*_3_ = 100. Apparently, removing the particles before their natural detachment time 1/*k*_3_ leads to a stronger disruption of the system.

The periodic removal and reattachment always causes temporary declustering coincident with the stepping burst. Interestingly, when Δ*t* < 1/*k*_3_, the subsequent clustering is stronger than that in the absence of the detachment/reattachment step (Figures 6 and 8). Clustering is therefore sped up by this mechanism. In the quasi-steady state, the average cluster size is however smaller than in the absence of the detachment/reattachment step (Figure 8).

### Finite Number of Particles

The constant attachment rate *k*_1_^′^ assumed in the previous section is equivalent to a non-depletable pool of particles available for binding. Here, we consider a situation where the total number of particles *e*_0_ is finite, the number of free particles *n*_e_ cannot be approximated by *e*_0_, and the actual attachment rate *k*_1_^′^ depends on the number of free particles *n*_e_: *k*_1_^′^ = *k*_1_*n*_e_. Of particular interest are the configurations with the total number of particles being of the same order of magnitude as the number of attachable sites on the chain.

In contrast to the previous section, no particles were attached to the chain at the start of the simulation. The observed kinetics therefore reflect the interplay of the attachment/detachment kinetics from an initial non-equilibrium state and the crowding effects due to the particles stepping along the chain.

The step rate in this section is expressed as the step rate per attachable site *j*_a_ = *j*/*u* and therefore represents the reduction of the step rate relative to the hypothetical case when all binding sites are occupied and the particles make steps unhindered.

In the following, we focus on the differences in kinetics compared to the results presented in the previous section caused by the finite particle number and the different initial condition.

#### Initial Burst of the Step Rate

**Figure 9:**
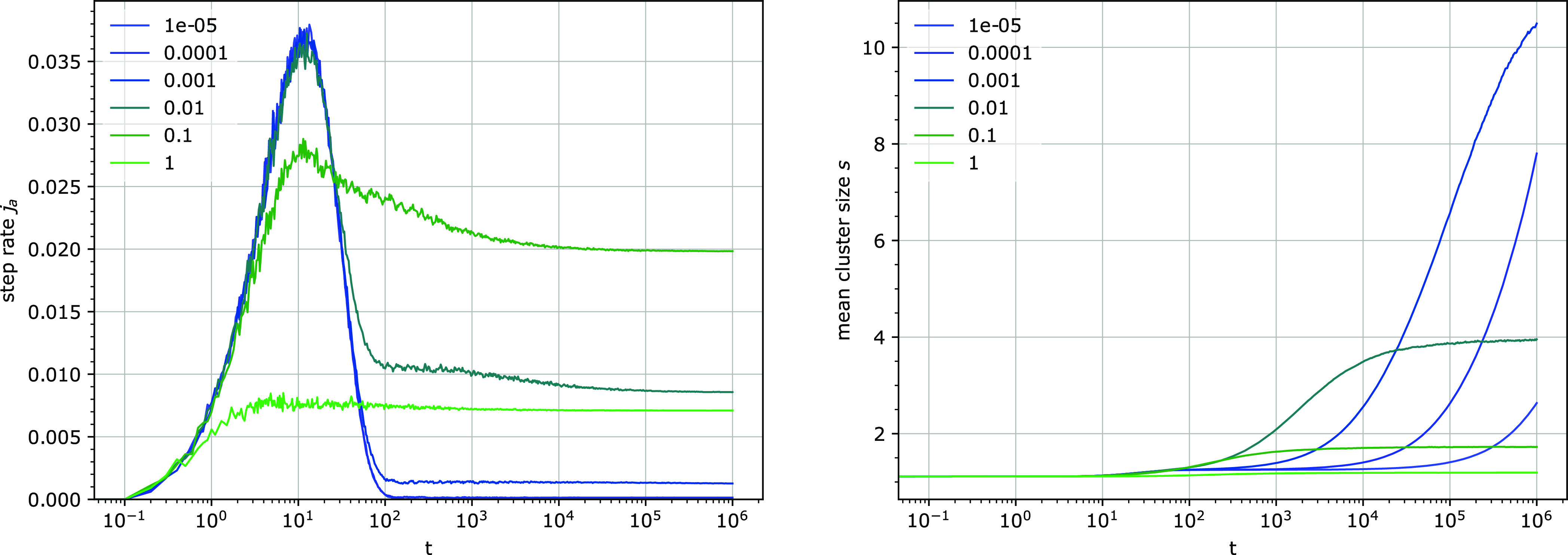
Step rate per attachable site *j*_*a*_ (left) and the mean cluster size *s* (right) for several detachment rates *k*_3_. Other parameters: *k*_1_ = 0.0001, *u* = 0.1, *e*_0_ = 100.

Depending on the model parameters, an initial burst in the step rate is observed, followed by a gradual decrease toward the steady state (Figure 9). In some simulations, a second phase of the decrease of the step rate was observed. The initial rise of the burst is governed by the binding of particles onto the initially empty chain. The subsequent fall of the step rate *j*_a_ is caused by the increasing blockage of the stepping particles, similar to the results in the previous section. The dependence of the position and amplitude of the burst depends on all model parameters in a complex way.

**Figure 10:**
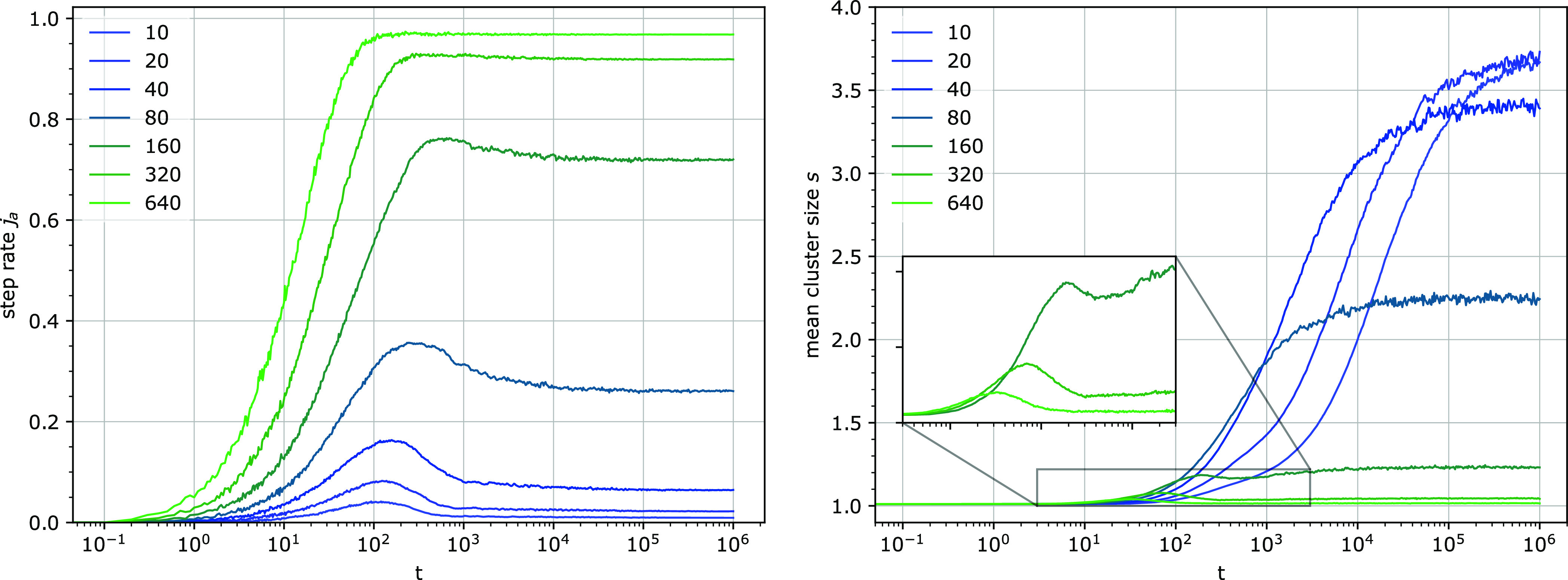
Step rate *j*_a_ (left) and the mean cluster size *s* (right) for several particle concentrations. The total number of binding sites *uN* is 82. Other parameters: *k*_1_ = 0.0001, *k*_3_ = 0.001, *u* = 0.01.

The presence of the burst depends on the total number of particles relative to the total number of binding sites (Figure 10). When the number of particles is smaller than the number of binding sites, the burst is prominent and the step rate at long times is clearly smaller than the burst maximum. With higher particle numbers, the steady-state step rate increases, making the burst less discernible or even absent. For some parameter values, even in the limit of *e*_0_ → ∞, a small burst can be observed; however, its amplitude is low compared to the steady-state value.

**Figure 11:**
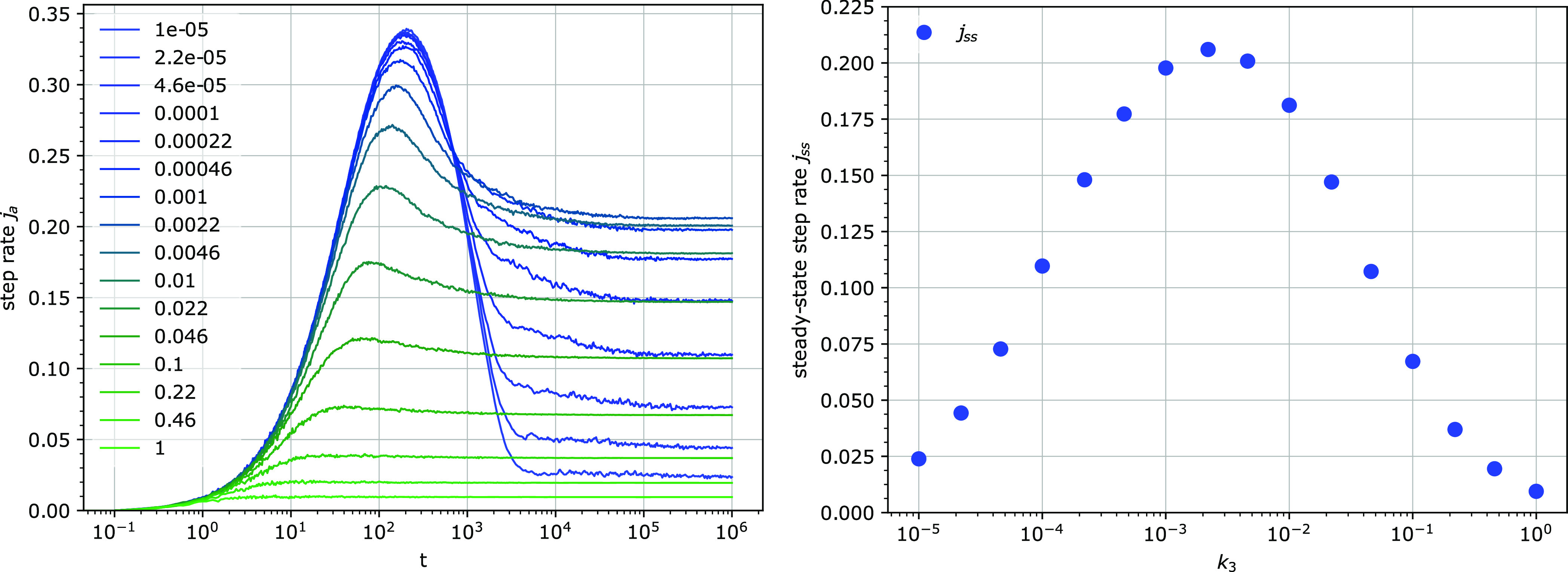
Temporal dependence of the step rate *j*_a_ (left) and the steady-state value *j*_ss_ of the step rate *j*_a_ (right) for different detachment rates *k*_3_. Other parameters: *k*_1_ = 0.0001, *u* = 0.014, *e*_0_ = 100.

Investigating the kinetics with dependence on the detachment rate *k*_3_ revealed that there is an optimal detachment rate for which the step rate *j*_a_ in the steady state is maximized (Figure 11). A higher than optimal detachment rate leads to a lower occupancy of binding sites and therefore to a lower steady-state step rate per binding site. A lower detachment rate enhances crowding because of blocking by unoccupied binding sites for which no free particle is available. This has important implications for the applications of this model since the existence of the optimal detachment rate in the context of cellulose hydrolysis has been suggested before^39^ and is also supported by experiments.^40,41^ This behavior has previously been interpreted as a demonstration of the Sabatier principle in the heterogeneous catalysis of cellulose by cellulases.^42^

Although the simulations were performed on a single chain, rescaling of the parameters makes it possible to reinterpret a set of data as simulations with a constant total particle concentration and a variable chain concentration. This enables a comparison with the experimental data of cellulose hydrolysis by cellulases.^31,43^ To do this, the parameters *e*_0_ and *k*_1_ were scaled by a factor α in the following way: *k*_1_ → α*k*_1_ and *e*_0_ → *e*_0_/α. The parameter α is then proportional to the chain (substrate) concentration. When the step rate in this case is expressed as the step rate per particle (total) *j*_e_ = *jN*α/*e*_0_, it becomes directly proportional to the experimentally observable hydrolysis rate at a constant cellulase concentration.

**Figure 12:**
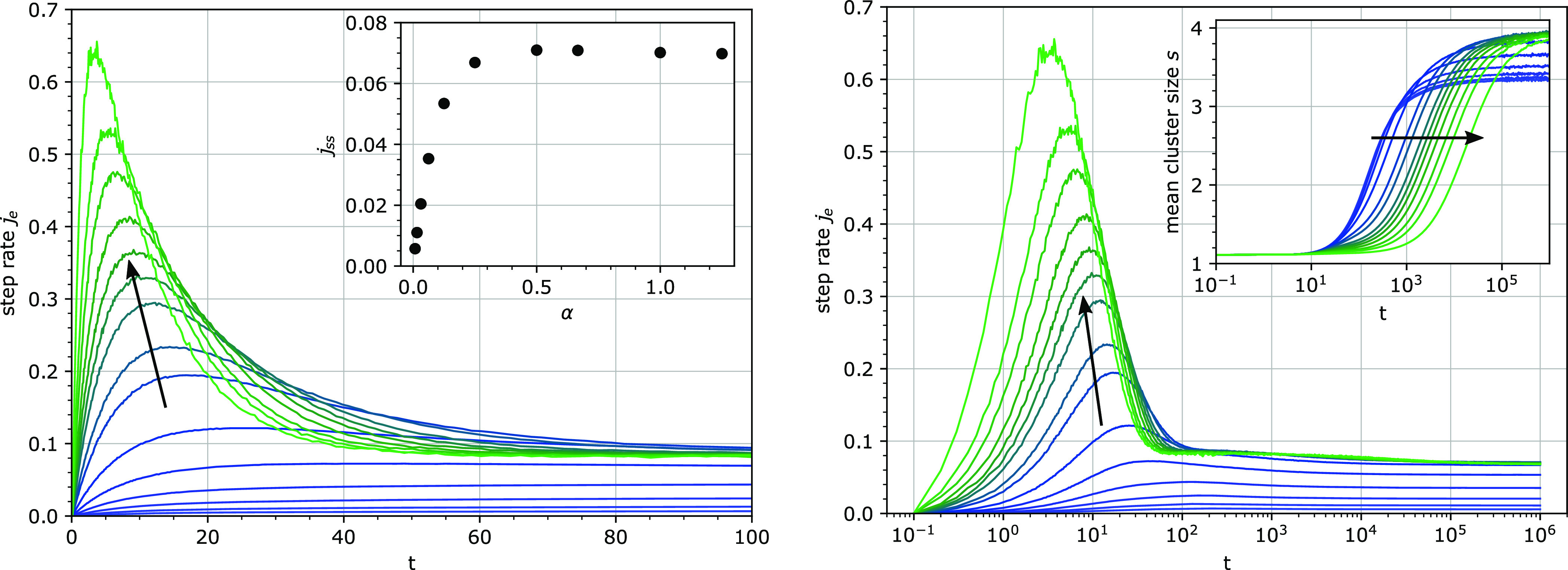
Step rate *j*_e_ per particle (total) for different chain concentrations α on logarithmic (left) and linear (right) time scales. The inset on the left shows the steady-state value *j*_ss_ of the step rate *j*_e_. The inset on the right shows the increase of the mean cluster size *s* with time. The parameter α, expressing the relative chain concentration, was varied from 0.00781 to 8.33, and its increase is indicated by the direction of the arrow in the plots. Other parameters: *k*_1_ = 0.0001, *k*_3_ = 0.01, *u* = 0.1, *e*_0_ = 100.

Varying the chain concentration over several orders of magnitude leads to the following observations (Figure 12): At low chain concentrations, the burst in the step rate is not discernible, and the step rate increases to its steady-state value. At increasing chain concentrations, a clear burst appears and its amplitude rises. With a higher chain concentration, the burst becomes narrower in time and its maximum appears at shorter times. At long times, the steady-state step rate shows a saturation behavior with increasing chain concentration α. Remarkably, all these features can be found in the experimental data of cellulose hydrolysis.^31,43^ Clustering of binding sites at very long times appears to be stronger with increasing chain concentrations, but the growth of clusters is considerably slower.

**Figure 13:**
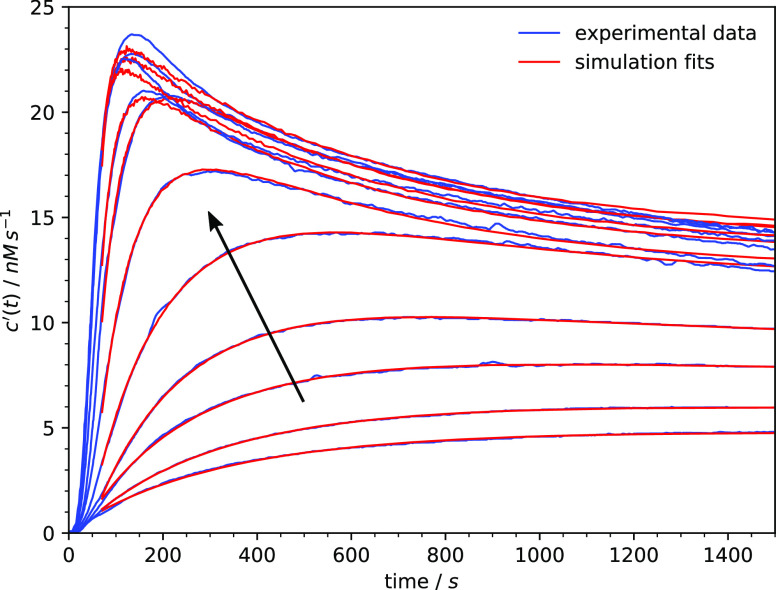
Fits of the simulation results to the experimental data from ref (31), describing the evolution of the rate of cellulose hydrolysis for different concentrations of the substrate. The substrate concentration increases from 1.5 to 110.9 μM in the direction of the arrow.

To demonstrate the agreement of the model with experimental data, we attempted to fit the simulation results to the published data of the time-dependent rate of cellulose hydrolysis for different concentrations of the substrate^31^ (Figure 13). The commonly used iterative fitting procedure, where the model is evaluated many times with different parameters until it converges to the data, was not possible in this case because of the long time needed to perform a single simulation. Therefore, we first fixed several simulation parameters common to all data curves (*k*_1_ = 8.3 × 10^–5^, *k*_3_ = 0.0138, *u* = 0.01, and *e*_0_ = 200) and then manually adjusted the substrate concentration parameter α and the amplitude to match each individual data set. Although this procedure does not guarantee that the fits are optimal or the parameters unique, the agreement with the data is very good, especially in the lower range of substrate concentrations.

#### Clustering of Binding Sites

As in the previous section, an increase in the mean cluster size *s* over different time scales was observed (Figure 9). One or often two phases could be discerned. In some situations, partial declustering, coinciding with the hydrolysis burst, takes place (Figure 10). Even though it has not been investigated in detail, it is thought to be related to the fact that the binding equilibrium due to the attachment/detachment kinetics has not been fully reached yet at the time when declustering occurs (eq 5).

#### Periodic Removal of Particles

**Figure 14:**
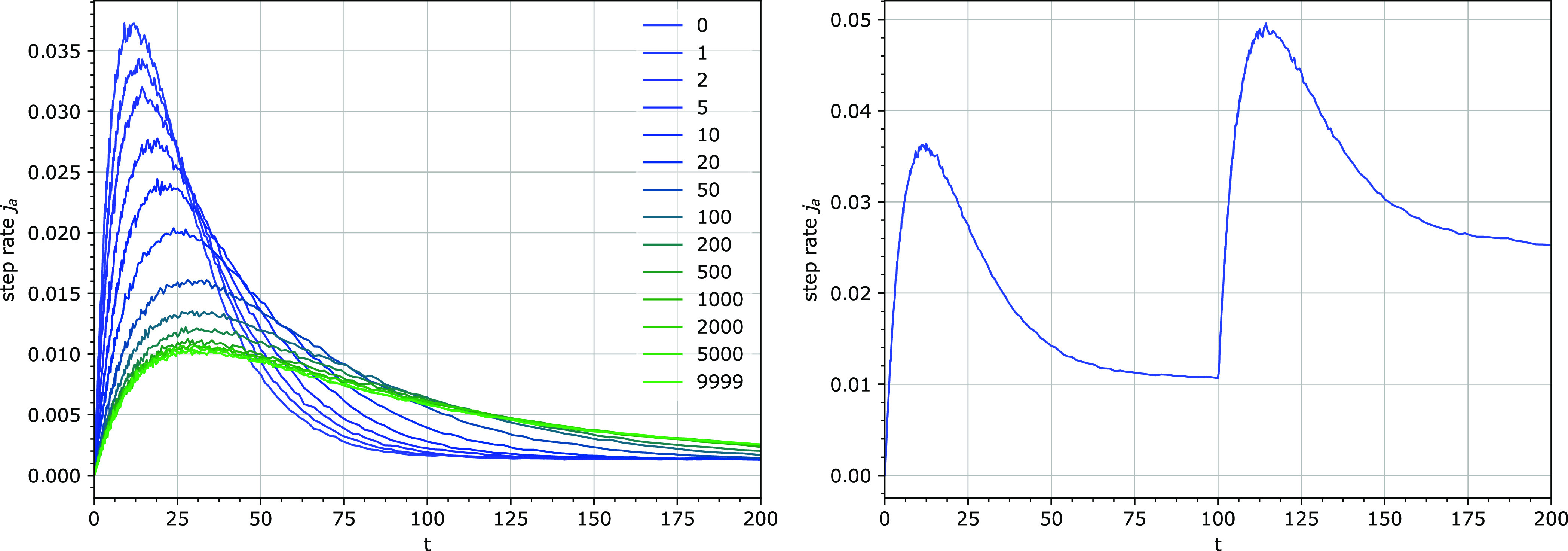
Left: the shapes of the bursts in the step rate *j*_a_ after different numbers of particle detachment cycles. Simulation parameters: *k*_1_ = 0.0001, *k*_3_ = 0.001, *u* = 0.1, *e*_0_ = 100. Right: the appearance of the second burst in the step rate after increasing the total number of particles *e*_0_. The simulation was started with *e*_0_ = 100 and at time *t* = 100, the total particle number *e*_0_ was increased by adding 100 free particles. Other parameters: *k*_1_ = 0.0001, *k*_3_ = 0.01, *u* = 0.1.

The simulations with periodic removal of particles, but no immediate reattachment, exhibited similar kinetics to the simulations with the excess of particles (Figures 6–8): bursts in step rates with progressively lower amplitude and longer duration, approaching a constant shape at a quasi-steady state (Figure 14). The step rate bursts were accompanied by transient declustering.

A similar burst in the step rate is produced when the total number of particles is increased by adding free particles at one time point, without affecting the bound particles in any way (Figure 14). This models the addition of an enzyme to a running hydrolysis reaction and has been shown experimentally to produce an analogous burst in the hydrolysis rate.^31,38,43,44^

## Discussion and Conclusions

### General Properties of the CB Model

The introduced CB model can be viewed as an extension of TASEP-LK. The modification—the distinction between binding and non-binding sites—leads to two immediately obvious differences. First, the fraction of binding sites *u* sets a limit to the maximum fraction of total sites that can be occupied by particles. In TASEP-LK, all sites can be occupied in the limit of *k*_1_^′^ ≫ *k*_3_. Second, the stepping of particles is blocked by binding sites on their right (in the direction of transport) regardless of their occupancy. This causes stronger blocking at low occupancies. Empty binding sites are stronger blockers than occupied sites in a sense that an attached particle can temporarily unblock a particle that it is blocking by making a step forward itself. These differences have important consequences for both the relaxation kinetics from the initial state and for the steady state reached in the limit of long times.

In this work, we are interested in the general crowding effects along the chain; therefore, we choose the periodic boundary condition to avoid any influence of the boundaries. We are mainly concerned with the relaxation kinetics from the initial state toward the stationary state and with their relation to the formation of the clusters of binding sites as time proceeds. In the published studies of TASEP-LK, the focus has been mostly on the stationary state in a system with open boundaries, where the boundary conditions are determinant for the properties of the stationary state.^17^ Nevertheless, there are several studies of TASEP-LK under conditions similar to those applied here,^18−20^ which allow a comparison with the results of the presented model.

Because the attachment and detachment kinetics is independent of the transport of particles along the chain and of the occupancy or type of neighboring sites both in TASEP-LK and in the CB model, the fraction of the occupied attachable sites when the binding equilibrium is reached is also the same. The relaxation kinetics of the particle density *f*(*t*) is exponential with equal time constants in both models^19^ (eq 6).

In TASEP-LK, the maximum steady-state current *j* is reached when half of the sites are occupied,^18^ and when expressed per attached particle, the steady-state current *j*_p_ decreases linearly with occupancy. In the presented CB model, the situation is more complex as the steady-state step rate *j*_p_ is not determined fully by the fraction of occupied binding sites *f* but depends directly on the rates *k*_1_^′^ and *k*_3_ (Figures 3 and 5). A slower particle exchange kinetics promotes formation of larger clusters of binding sites, decreasing the step rate *j*_p_. The relationship between the mean cluster size and the step rate is however not trivial (Figure 5). A more detailed investigation considering also the distribution of cluster sizes might deepen the understanding of this aspect.

The relaxation kinetics of the particle current *j*(*t*) in TASEP-LK approaches the steady state exponentially, with a time constant similar to the relaxation of the particle density *f*(*t*). For certain initial conditions (no attached particles) and certain parameter values, the relaxation kinetics can also exhibit a maximum before decreasing toward the steady state.^20^ Here, the relaxation of the step rate *j*_p_ is affected by the formation of clusters of binding sites over time. The kinetics has more phases and is stretched in time, slower than exponential, which is particularly evident in the increase of the mean size of clusters at long times (Figures 3 and 4). The relaxation kinetics is influenced by the initial distribution of binding sites and the initial degree of clustering, as observed in the simulations with detachment and reattachment of particles (Figures 6 and 14). The individual detachment and reattachment cycles can be understood as independent simulations with different initial conditions with respect to the distribution of binding sites.

The presented results show that the distinction between binding and non-binding sites leads to a much richer behavior compared to that of the original TASEP-LK model.

### Model as a Description of Cellulose Hydrolysis

The existing simulation models of cellulose hydrolysis by processive cellulases usually incorporate a high level of detail in the description of the structure of both the enzymes and the substrate and in the individual interaction steps. The enzyme molecules are characterized not only by their size but also the shape^32,35^ and sometimes even by their internal structure, resolving the catalytic domain, the carbohydrate binding module, and the linker connecting the two subunits.^33,34^ The substrate is represented as a 2D assembly of linear chains, a 3D microfibril with a particular arrangement of chains,^33,34^ or a more complex structure with domains of different properties.^35^ In some situations, the particular shape of the substrate and its evolution during the course of the reaction are decisive for the observed kinetics.^32^ Different states of the enzyme in interaction with the substrate are considered; every state may exhibit a different type of transport along the substrate (diffusional or processive), and various transitions between the states, in addition to adsorption and desorption, are a part of the model.

Consequently, the models usually contain many parameters, making the characterization of the model behavior in the large parameter space impractical. It may be difficult to elucidate which detail of the model is determinant for the overall model behavior, and what feature of the model is less or not at all relevant.

In this work, we choose to focus on the role of crowding of enzyme particles on the substrate chain by excluding all but the essential detail and thus keeping the model minimal. The events in the CB model may then effectively represent more than one real event. The attachment represents all steps between the actual attachment and the start of processive hydrolysis, including threading of the chain. The detachment represents all steps between the stop of processive hydrolysis and the physical detachment from the substrate, including decomplexation. One model step of the enzyme along the chain may in reality correspond to more hydrolysis steps.

Despite these simplifications, the inclusion of particle interactions that leads to crowding results in a model that can reproduce a broad range of experimental observations: At short times, a hydrolysis burst appears (Figure 9) and a similar activity burst is observed upon addition of more enzymes (Figure 14). The temporal profile of the burst with increasing substrate concentration exhibits faster rise, its maximum is reached earlier, and its value is higher relative to the steady-state hydrolysis rate; at low substrate concentrations, no clear maximum is attained (Figure 12). When the detachment rate *k*_3_ is varied, a maximum in the steady-state hydrolysis rate for a particular detachment rate is reached (Figure 11), as suggested in the literature.^40^ The steady-state hydrolysis rate shows a saturation behavior with increasing substrate concentration (Figure 12). The detailed view of the simulations reveals the presence of clusters of attached particles that can be released in a micro-burst, as observed directly in AFM experiments^27^ (Figure 2).

The results of the simulations are also in agreement with a simple analytical three-state model, which assumes the existence of an active, hydrolyzing bound state and an abstract inactive bound state.^45^ The analytical model described the experimental data best when assuming that the initially free particle first binds to the active state and later enters the inactive state. The CB model introduced here offers a concrete picture as to the nature of the active and inactive states. The binding sites are initially minimally clustered; a particle that binds to the chain is likely to be free to perform steps, that is, is active. After some time, the particle is going to hit a binding site, becoming blocked. The blocked state corresponds to the inactive state in the analytical model.

Due to its simplicity, the CB model still exhibits a couple of features for which there is lack of experimental support. Several sets of simulation results show that the crowding effect on the hydrolysis rate can be diminished by adding an excess of enzyme. This leads to the increase of the occupancy of the binding sites toward saturation, effectively unblocking the crowded enzyme molecules. The effect can be seen in Figure 5, where the steady-state step rate *j*_p_ can be brought toward its maximum value of *k*_2_(1 – *u*) by strongly increasing the attachment rate *k*_1_^′^, regardless of the detachment rate *k*_3_. As seen in Figure 10, the increase of the total number of enzyme particles *e*_0_ well above the number of the binding sites on the chain not only eliminates the initial hydrolysis burst but also brings the step rate *j*_a_ close to its maximum value corresponding to unhindered hydrolysis. Although this effect is not very realistic, it does not invalidate the model but rather shows that the model is not complete and has its limits. The evidence from published experimental data provides hints to potential extensions of the CB model that might address this issue,^26,46,47^ for example, including unproductive binding, where the interaction of the enzyme with the chain does not permit hydrolysis, possibly because the chain is not threaded. Such a bound enzyme would still block the processive motion of other enzyme particles. Another possibility is the modification of the interactions between particles and chain ends that takes into account the difference in size of an enzyme molecule and the binding site, as already used in the earliest application of TASEP on enzyme sliding along a nucleic acid.^7^

In experiments performed over long times, a gradual decrease of hydrolysis activity over time scales much longer than the initial activity burst is commonly observed.^44,46^ In the CB model, the steady state is often reached relatively fast after the initial burst, and if there is any decrease of the step rate occurring later, it is relatively small. Interestingly, coupled to this small step rate decrease at long times is a significant increase in clustering (Figures 3, 4, 8, and 10). This raises the possibility that modifying the model by inducing a stronger coupling between the degree of clustering and the step rate might lead to a step rate decrease also over long time scales, as known from experiments.^44,46^ Nevertheless, it should be noted that there are other factors thought to contribute to the long-term effects in hydrolysis, such as changes in the substrate morphology and effects of substrate heterogeneity, that become apparent as a significant fraction of the substrate becomes hydrolyzed. These are, however, beyond the scope of the presented model, which effectively reduces the substrate to a single chain and does not deal with the substrate structure on a larger scale.

In the search for the explanation of the decrease of hydrolysis rate in the course of cellulose hydrolysis, a distinction between substrate effects and enzyme effects is often made.^31,47,48^ The substrate effects are linked to the heterogeneity of cellulose with different fractions (e.g., crystalline vs amorphous) having different susceptibilities to hydrolysis, resulting in evolving substrate composition and properties as hydrolysis progresses. Alternatively, the substrate effects can be described as depletion of productive binding sites in the course of hydrolysis. The enzyme effects usually involve some type of inactivation, reversible or irreversible, either by adsorption on noncellulotic components (lignin) or by nonproductive binding to cellulose.

The kinetics of the presented CB model shows that the decrease in the step rate is accompanied by an increase in the mean cluster size *s*. Although the relationship between the step rate and the mean cluster size is not straightforward, it is intuitive to expect that larger clusters of binding sites lead to a lower step rate: in a cluster, only one, the rightmost site is not blocked; therefore, with the mean cluster size *s*, only the fraction 1/*s* of all binding sites is not blocked and can contribute to the step rate if occupied by a particle. This reasoning suggests that the retardation of the step rate in the CB model clearly falls into the category of substrate effects. The simulations with the detachment and reattachment of particles show, however, that the particle occupancy of sites within a cluster plays an important role, and the effect cannot be attributed solely to the substrate.

After a long time, most particles are bound to blocked sites within a cluster. Detachment and random reattachment of particles obviously does not change the distribution of the binding sites on the chain at that very moment. It is the distribution of particles within a cluster that changes at this step. While before the particle detachment the rightmost cluster site is less likely to be occupied (if occupied, the particle would perform a step, separating itself from the cluster), after the particle reattachment, it has the same probability of being occupied as all other binding sites. This is the reason for the subsequent increase in the step rate and decrease in the mean cluster size (Figures 6 and 14). Both effects are transient but can appear at any time after the beginning of hydrolysis, even in the steady state, when larger clusters are formed and the initial condition is forgotten (Figure 14). The effect is therefore independent of the assumption of randomly distributed, that is, rather dispersed, binding sites made in the simulations. From this point of view, the particles being blocked is the cause of the decreased step rate. The addition of new particles instead of detachment of the already present particles leads to a similar effect by the same mechanism (Figure 14).

Following this picture, there is a clear dependence between the effects of substrate structure and enzyme blocking on the step rate: while particle stepping contributes to the growth of clusters, larger clusters increase the population of blocked particles. The intervention by detachment/reattachment transiently reverses this process: freeing the particle from its blocked state and giving it a chance to bind to the rightmost (not blocked) cluster site increases the step rate and promotes cluster disassembly. The substrate and enzyme effects within the CB model cannot therefore be separated from each other.

The detachment/reattachment cycles performed periodically can increase the average step rate (Figure 6), and if realized in practice, it could form a basis of a strategy to enhance the efficiency of cellulose hydrolysis. This view is supported by experimental observation of a hydrolysis burst after addition of new enzyme^31,38,43,44^ and the partial recovery of hydrolysis rate after the enzyme removal and the restart of hydrolysis.^46,48^

Although only one type of enzyme was considered in this work, in practice, the cellulase Cel7A is usually not acting alone but is present together with other cellulases, which may attack the cellulose chain in a different way. These include exocellulases hydrolyzing the oriented cellulose chain from the opposite end than the exocellulase Cel7A and endocellulases, which hydrolyze also the bonds in the middle of the chain, and so create new chain ends, that is, new binding sites for exocellulases. The combined action of exo- and endocellulases is expected to affect the density of the binding sites (the parameter *u*, held constant in this work) and therefore also to alter the degree of site clustering and its effects on the overall enzyme kinetics. Modeling of cellulose hydrolysis by various combinations of different enzymes will be a natural extension of the presented work and is expected to contribute to the understanding of the experimentally observed synergies between different cellulases.

In conclusion, the proposed CB model shows that many features of cellulose hydrolysis catalyzed by processive cellulases can be explained by blocking of the transport of cellulases along the cellulose chain by other binding sites, whereby dynamic assembly of the binding sites into clusters during the course of the reaction and the occupancy of the binding sites within the cluster modulates the overall reaction rate. Importantly, it is not necessary to assume the existence of an additional non-productively bound state or irreversible enzyme binding.
